# Development and Evaluation of a System for the Semi-Quantitative Determination of the Physical Properties of Skin After Exposure to Silver Nanoparticles

**DOI:** 10.1186/s11671-020-03421-x

**Published:** 2020-09-29

**Authors:** Hong Tao, Kazuya Nagano, Ikkei Tasaki, Tian-qi Zhang, Takuya Ishizaka, Jian-Qing Gao, Kazuo Harada, Kazumasa Hirata, Hirofumi Tsujino, Kazuma Higashisaka, Yasuo Tsutsumi

**Affiliations:** 1 Graduate School of Pharmaceutical Sciences, Osaka University, 1-6 Yamadaoka, Suita, Osaka 565-0871 Japan; 2 Graduate School of Medicine, Osaka University, 2-2 Yamadaoka, Suita, Osaka 565-0871 Japan; 3 Institute of Pharmaceutics, College of Pharmaceutical Sciences, Zhejiang University, 866 Yuhangtang Road, Hangzhou, 310058 PR China; 4 The Center for Advanced Medical Engineering and Informatics, Osaka University, 1-6, Yamadaoka, Suita, Osaka 565-0871 Japan

**Keywords:** Single particle inductively coupled plasma-mass spectrometry, Silver nanoparticles, Dermal toxicity

## Abstract

In order to ensure the safe usage of silver nanoparticles (nAgs) in cosmetics, it is necessary to reveal the physical properties of nAgs inside the skin, as these properties may change during the process of percutaneous absorption. In this study, we aimed to establish an analytical system based on single particle inductively coupled plasma mass spectrometry (sp-ICP-MS) to determine the physical properties of nAgs in the skin. First, we optimized a pretreatment method for solubilizing the skin samples and then showed that most of the nAgs were recovered by sodium hydroxide treatment while remaining in particle form. For separating the skin into the epidermis and dermis, we screened several conditions of microwave irradiation. The sp-ICP-MS analysis indicated that the application of 200 W for 30 s was optimal, as this condition ensured complete separation of skin layers without changing the physical properties of the majority of nAgs. Finally, we evaluated the in vivo application by analyzing the quantity as well as the physical properties of Ag in the epidermis, dermis, and peripheral blood of mice after exposing the skin to nAgs or Ag^+^. Subsequent sp-ICP-MS analysis indicated that nAgs could be absorbed and distributed into the deeper layers in the ionized form, whereas Ag^+^ was absorbed and distributed without a change in physical properties. This study indicates that in order to obtain a comprehensive understanding of the response of skin following exposure to nAgs, it is essential to consider the distribution and particle size of not only nAgs but also Ag^+^ released from nAgs into the skin.

## Introduction

Recent technological advances in nanotechnology have accelerated the development of engineered nanoparticles (ENPs) that are particles smaller than 100 nm. Owing to their beneficial properties, such as enhanced tissue penetration and surface reaction, in comparison to micro- or larger-sized materials, ENPs are widely used in various products, including cosmetics, food, and medicine [1–3]. For example, silver nanoparticles (nAgs), one of the most common types of ENP, are incorporated into cosmetics because of their antibacterial properties resulting from a steady release of silver ions (Ag^+^) [4, 5]. However, the unique physicochemical properties associated with the small particle size of nAgs can be hazardous. It is known that these particles may disrupt otherwise impenetrable barriers, such as the blood-brain barrier, and induce inflammation [6]. In addition, some studies have reported that ENPs can penetrate the skin barrier [7–9]. Therefore, to determine the safety of continuous use of these particles, it is important to understand the toxic effects associated with ENPs containing nAgs by investigating the dynamics of these particles within tissues, such as the skin.

In order to ensure safety, it is indispensable to understand the possible risks associated with using ENPs, which involves the integrative concepts of “hazards” (potential toxicity) and “exposure conditions.” While the hazards of ENPs have been analyzed worldwide, only a few studies have examined the conditions pertaining to the exposure to ENPs [10]. In particular, it has been reported that nAgs and Ag^+^ can change their physical properties within the body. For example, the ionization of nAgs results in the formation of nAgs with a smaller particle size and the release of Ag^+^ [11]. Conversely, nAgs having small particle size can be detected in the intestinal epithelium of rats following oral administration of silver acetate [12]. Furthermore, we have recently reported that compared to smaller nAgs and Ag^+^, larger nAgs are more readily found in the breast milk of lactating mice exposed to those nAgs [13]. Therefore, nAgs may change its physical properties in the body, which in turn leads to a change in kinetics. Thus, in order to understand the risks involved, it is necessary to evaluate the physical properties, such as particle size, of these particles, and to distinguish between these particles and ions in the body.

In this respect, we applied single particle inductively coupled plasma mass spectrometry (sp-ICP-MS), which introduces a maximum of one particle into the analyzer per dwell time. It is an effective method that can be used to determine particle size by analyzing peak intensity and particle concentration via peak rates. Particles and ions can be distinguished by analyzing both peak and background signals [14]. We have previously optimized a pretreatment method for sp-ICP-MS in biological samples to semi-quantitatively determine the physical properties of ENPs in various organs, such as the liver, heart, lungs, kidneys, and the spleen [15].

The skin comprises the epidermis, including the stratum corneum (SC), and the dermis, containing blood vessels, lymphatic vessels, and nerves [16]. Therefore, the entry of ENPs into each skin layer may induce toxicity in varying degrees. For example, the distribution of titanium dioxide nanoparticles in human skin keratinocyte cells of the epidermis may stimulate the production of reactive oxygen species [17]. Moreover, in hairless mice, dermal exposure to titanium dioxide nanoparticles for 60 days not only led to pathological changes, such as a thinner dermis due to local toxicity, but also to pathological changes in the liver, such as liquefaction necrosis due to systemic toxicity that spread via blood vessels in the dermis [18]. In addition, the biological responses in each layer may also vary depending on physical properties, such as particle size, and differences between particles and ions [19]. In order to understand the safety of using nAgs, it is necessary to understand the physical properties and biodistribution of nAgs after it has been exposed to skin.

In order to resolve this particular issue, an approach that can pretreat the skin and separate its layers without causing any losses during recovery or changes in the physical properties of ENPs is needed. However, such an optimal approach for the skin has not been devised.

In this study, we optimized a pretreatment approach that semi-quantitatively determines the physical properties of nAgs, a model ENP, in each layer of the skin via sp-ICP-MS, and subsequently evaluated its effectiveness in vivo.

## Methods

### Mice

Slc:ICR mice (female, 8 weeks old) were purchased from Japan SLC (Shizuoka, Japan). Mice were housed in a room with the following light-dark cycle: lights on at 8 am and off at 8 pm. Food and water were made available in the form of food pellets and a water supply system located on top of the cage. All experimental protocols were performed under conditions approved by the animal research committee of Osaka University, Japan.

### nAgs and Ag^+^

Suspensions of citrate-ligand-capped nAgs with diameters of 100 nm (nAg100) were purchased from nanoComposix (San Diego, CA, USA) in the form of stock dispersions (1 mg/mL). Silver nitrate (AgNO_3_) was purchased from Wako Pure Chemical Industries (Osaka, Japan), also in the form of stock dispersions (1 mg/mL). RM8013, used as a standard for calculating transport efficiency, was purchased from the National Institute of Standards and Technology (Gaithersburg, MD, USA). Each type of nanoparticle was sonicated for 10 min prior to use. Nanoparticles and ions were also vortexed for 10 s prior to use.

### Reagents

Sodium hydroxide (NaOH, 0.1 mol/L) was purchased from Nacalai Tesque Company (Osaka, Japan) and nitric acid (HNO_3_, 70%) was purchased from Kanto Kagaku Chemical Industries (Tokyo, Japan). Phosphate-buffered saline (PBS), pH 7, was prepared.

### Optimization of Pretreatment Methods

The epidermis and the dermis of each mouse were separated, mixed with PBS (*w/v* ratio of 1:10), and homogenized. The homogenate was mixed with a 100 ng/mL nAg100 solution. The mixture was then treated with one of the following reagents at a *v/v* ratio of 1:1; 0.1 mol/L NaOH, 70% HNO_3_, or PBS. The samples were incubated for 3 h at 37 °C and subjected to sp-ICP-MS.

### Skin Separation via Microwave Irradiation

Each mouse was euthanized with isoflurane (Wako), after which a 2 cm^2^ (2 cm × 1 cm) dorsal skin sample was excised using surgical scissors and tweezers. Special care was taken to prevent tissue damage during the excision procedure. A microwave oven (RE-SW-20-H, Sharp, Japan) was used to irradiate the skin at a frequency of 2450 MHz. Skin samples were placed on a plate and put in the center of the microwave oven. In accordance with testing requirements, skin samples were irradiated for 10, 30, and 60 s at 200, 600, and 900 W, respectively. Following irradiation, the samples were rapidly removed and each epidermis and dermis were quickly separated by gentle scraping with surgical tweezers. A 100 ng/mL nAg100 solution was used for the analysis of the recovery rate and the average diameter of nAg after the irradiation (200 W, 30 s).

### Transdermal Administration of nAg100 and Ag^+^

Nine-week-old female Slc:ICR mice were divided into 6 groups of 3 mice per group, according to their body weight. The mice were anesthetized using isoflurane. The hair on their backs was shaved with a hair clipper (Panasonic®, Osaka, Japan) and a hand razor (Gillette®, Germany). Next, nAg100 and Ag^+^ (20 μg/cm^2^) were directly applied to a surface area of 2.25 cm^2^ (1.5 cm × 1.5 cm) of dorsal skin and covered tightly with non-absorbent plastic film. A piece of gauze of the same size was placed on the plastic film. A self-adhesive elastic bandage was used to cover the gauze by wrapping around the skin and the skin was left covered for 5 days.

### Pretreatment of Skin Samples and Blood

Five days after treatment, peripheral blood from the retro-orbital venous plexus and dorsal skin was collected for analysis. Next, 2.25 cm^2^ (1.5 cm × 1.5 cm) dorsal skin samples were excised using surgical scissors and tweezers. Special care was taken to prevent tissue damage during excision. SC layers were sequentially removed using 2-cm pieces of adhesive tape (Scotch®, 3M), prior to separating the epidermis from the dermis. The pieces of tape were pressed on the treated area of the dorsal skin after which constant pressure was applied for 10 s. Twenty pieces of tape were required to remove the entire SC of each mouse. Next, the dermis and epidermis were separated with microwave irradiation and homogenized with PBS (*w/v* ratio of 1:10). Collected blood, as well as dermis and epidermis homogenates, were treated with 0.1 mol/L NaOH at a *v/v* ratio of 1:1 and incubated separately for 3 h at 37 °C. After incubation, the gross silver mass in the blood, epidermis, and dermis mixtures was analyzed with inductively coupled plasma mass spectrometry (ICP-MS). Quantification and assessment of the physical properties of nAgs and Ag^+^ were carried out using sp-ICP-MS.

### Measurement of the Gross Mass of Silver

In order to measure the total silver concentration in the blood, SC, epidermis, and dermis samples, an Agilent 7700x ICP-MS system (Agilent Technologies, Santa Clara, CA, USA) was used. The conditions under which the analysis was performed were as follows: RF power 1550 W; carrier gas 1.05 L/min Ar; and dwell time 100 ms. Measurements were repeated thrice in MS mode. Rhodium was used as the internal standard for Ag. The target elements of the ICP-MS analyses were ^103^Rh and ^107^Ag. Ag and rhodium standard solutions were obtained from Wako.

### sp-ICP-MS Analysis and Calculation

An Agilent 7700x ICP-MS (Agilent Technologies) was used for the sp-ICP-MS analysis. Analysis conditions were as follows: RF power 1550 W; carrier gas 1.05 L/min Ar; dwell time 10 ms; and analysis time 30 s. Single particle calculation tools—RIKILT published by Wagenen Food Safety Research (Wageningen University, Wageningen, Netherlands)—were used to calculate the particle size [20].

### Statistical Analysis

All statistical analyses were conducted using GraphPad Prism software version 5.0 for Macintosh (GraphPad Software, La Jolla, CA, USA). Statistical significance was set at *P* < 0.05.

## Results and Discussion

### Strategies for Constructing a Method to Determine the Quantity and Physical Properties of nAgs in Each Skin Layer

In order to determine the quantity and physical properties of nAg in each skin layer, it is necessary to completely solubilize the skin samples and prepare samples amenable to sp-ICP-MS analysis. It is also essential to separate the epidermis and the dermis, without losses during recovery, or changes in the physical properties of the nAg, because non-transdermally absorbed nAg can sometimes be eliminated during removal of the SC via tape stripping [21].

With regard to the solubilizing of the skin samples, we have previously reported that NaOH pretreatment is a technique that is optimal for the quantification as well as the analysis of the physical properties of nAg in animal tissues, such as the liver, heart, lungs, kidneys, and the spleen [16]. Therefore, NaOH pretreatment was applied to the skin samples used in this study.

Hydrothermal treatment, which leads to the softening of collagen fibers and enhanced enzyme digestion, which promotes separation at the epidermal-dermal junction (EDJ), is widely used to separate the skin into the epidermal and dermal layers [22]. Although these treatments efficiently separate skin layers, nAg in the layers may be ionized in an aqueous solution resulting in a change in its physical properties. It is reported that a short pulse of microwave irradiation allows the skin to be separated into epidermal and dermal layers through heat generation that disrupts the EDJ [23, 24]. Therefore, we applied microwave irradiation without incubation to the solution for a short time.

**Fig. 1:**
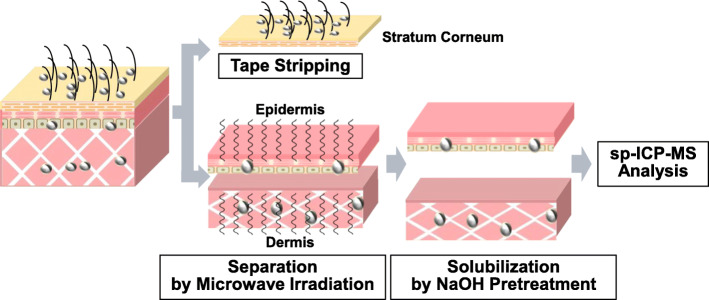
Strategy to determine the quantity and physical properties of nAg100 in each layer of skin tissue. In order to determine the quantity and physical properties of nAg100 in each layer of skin tissue it is necessary to (1) completely solubilize skin tissues and (2) separate the skin into epidermal and dermal tissues, without tissue loss during recovery or changes in the physical properties of nAg100. In this respect, we focused on (1) NaOH pretreatment and (2) microwave irradiation

Collectively, we proposed a strategy (illustrated in Fig. 1), which was validated by analyzing the recovery rates and physical property changes of nAg in each skin layer.

### Optimization of Pretreatment Methods for Detecting nAg100 in the Epidermis and Dermis

**Fig. 2:**
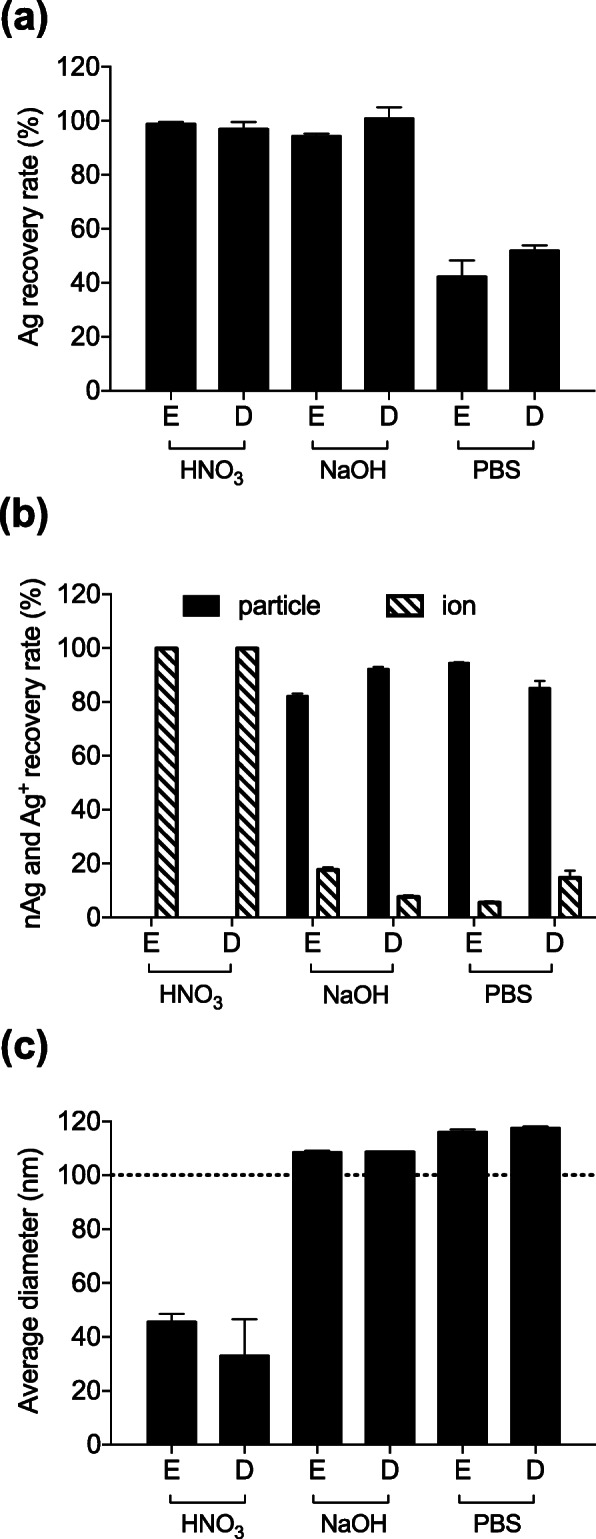
NaOH pretreatment is the optimal method for detecting nAg100 in dermis and epidermis. Two solubilizing reagents (HNO_3_ and NaOH) were screened as pretreatment solvents to lyse tissues. The epidermis (E) and dermis (D) homogenates were mixed with nAg100 to obtain a final Ag concentration of 100 ng/mL and treated with each solubilizing reagent at 37 °C. After 3 h, all samples were analyzed via ICP-MS and sp-ICP-MS. The **a** recovery rate of Ag, **b** nAg (black bars) and Ag^+^ (shaded bars) recovery rate, and **c** average particle diameters are presented. The dashed line represents the initial particle size. The data have been expressed as mean ± S.D. (*n* = 3)

We referred to our previous study in order to optimize a pretreatment method for solubilizing skin samples. Ishizaka et al. [15] reported that, among various kinds of solubilizing reagents, such as NaOH and HNO_3,_ NaOH pretreatment was optimal. Thus, we tested HNO_3_ and NaOH as acidic and alkaline solubilization reagents, respectively. The epidermis and dermis homogenates separated from the mouse skin samples were mixed with nAg100 to obtain a final Ag concentration of 100 ng/mL followed by treatment with each solubilization reagent at 37 °C. First, we evaluated the recovery rate of Ag following treatment with these reagents. ICP-MS analysis indicated that an almost 100% recovery rate was achieved by NaOH and HNO_3_ treatment only (Fig. 2a). Next, in order to evaluate changes in the physical properties due to each treatment, we analyzed the recovery concentrations of each particle and ion. sp-ICP-MS Analysis indicated that nAg100 was nearly completely ionized by HNO_3_. This suggested that HNO_3,_ as the acidic reagent, dissolved the particles and converted them into ions, as also reported in a previous study [15]. In contrast, a majority of the nAg100 treated with NaOH remained as particles, and not as ions (Fig. 2b). Thus, NaOH allowed the majority of the nAg100 to remain in a particle form. Finally, distributions of particle diameters were evaluated in order to analyze the physical properties in detail. HNO_3_ treatment changed the average particle diameter from 100 nm to 40 nm, which corresponded to the ionization of nAg100. Conversely, the average particle diameter after NaOH treatment was approximately 100 nm, corresponding to the initial particle size (Fig. 2c). This suggested that NaOH pretreatment was optimal for detecting nAg100 in mouse skin.

### Separation of Skin Samples into Epidermis and Dermis via Microwave Irradiation and Evaluation of Recovery Rates and Changes in Physical Properties of nAgs

**Fig. 3:**
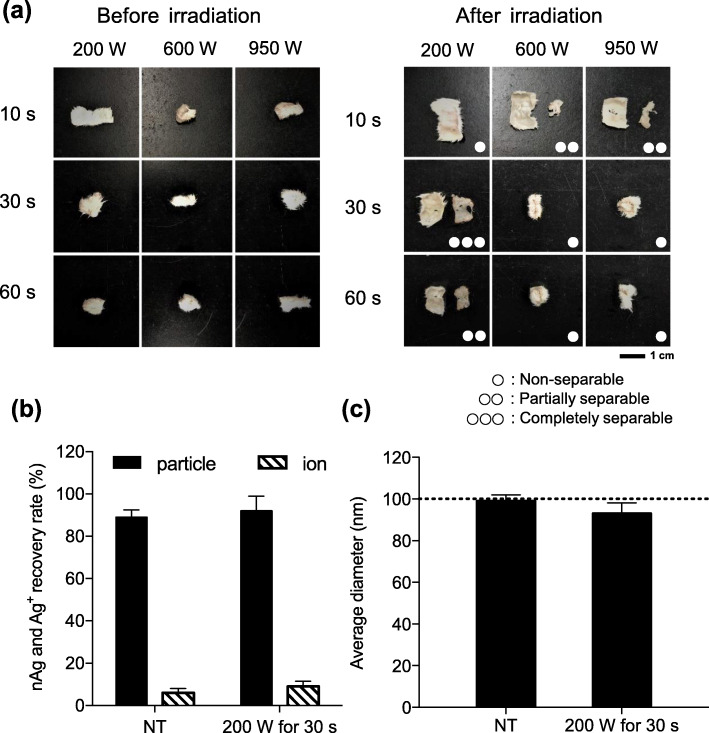
Screening and evaluation of conditions for separating the skin into epidermis and dermis by microwave irradiation. **a** Skin tissue was irradiated with microwaves under nine conditions and separated into epidermal and dermal tissues. Left and right panels show skin samples before and after irradiation, respectively. In the right panel, single, double, and triple white circles show non-separable, partially separable, and completely separable conditions, respectively. Scale bars: 1 cm. Skin homogenates were mixed with nAg100 to obtain a final Ag concentration of 100 ng/mL, and irradiated at 200 W for 30 s, and analyzed via sp-ICP-MS. **b** The nAg (black bars) and Ag^+^ (shaded bars) recovery rates and **c** average particle diameters are presented. The dashed line represents the initial particle size. The data have been expressed as mean ± S.D. (*n* = 3)

In order to separate skin samples into epidermal and dermal layers, we applied different conditions of microwave irradiation that were relevant to our study and previously reported to be successful [24] and compared their performance with respect to the separability of the skin and changes in the physical properties. It was observed that under the conditions of 200 W for 10 s, 600 W for 30 s, 600 W for 60 s, 950 W for 30 s, and 950 W for 60 s, the skin failed to separate into epidermal and dermal layers (Fig. 3a). Moreover, the skin was only partially separated into epidermal and dermal layers under the conditions of 200 W for 60 s, 600 W for 10 s, and 950 W for 10 s. In contrast, the skin was only completely separated into epidermal and dermal layers after irradiation at 200 W for 30 s. These results indicate that irradiation at 200 W for 30 s is optimal for skin layer separation.

In order to verify whether this microwave irradiation at 200 W for 30 s affected the physical properties of nAg100, we determined the physical properties of Ag in epidermal and dermal layers. The epidermis and dermis homogenates, each mixed with 100 ng/mL of nAg100, respectively, were lysed using NaOH and irradiated at 200 W for 30 s. Sp-ICP-MS analysis revealed that the majority of the nAg100 treated with microwave irradiation remained as particles, and so did those of the non-treated group (Fig. 3b). In order to analyze the physical properties in detail, particle diameter distributions were also evaluated. The average particle was almost 100 nm in diameter, which corresponded to the initial particle size (Fig. 3c). These findings suggest that microwave irradiation of skin samples at 200 W for 30 s is a promising approach for efficiently separating skin into epidermal and dermal layers without changing the physical properties of nAg100.

Collectively, these results indicate that we had successfully developed a system to semi-quantitatively determine the physical properties of nAg100 in each skin layer. Specifically, the method entails the following: (i) removal of non-transdermally absorbed nAg100 by removing the SC with the tape stripping method [21]; (ii) separation of the skin into epidermal and dermal layers via microwave irradiation; (iii) solubilization of the epidermis and dermis with NaOH treatment; and (iv) the semi-quantitative determination of the physical properties of Ag in each skin layer using sp-ICP-MS.

### Practical Evaluation by Determining the Quantity and Physical Properties of nAg100 and Ag^+^ in Mouse Skin Layers In Vivo

**Fig. 4:**
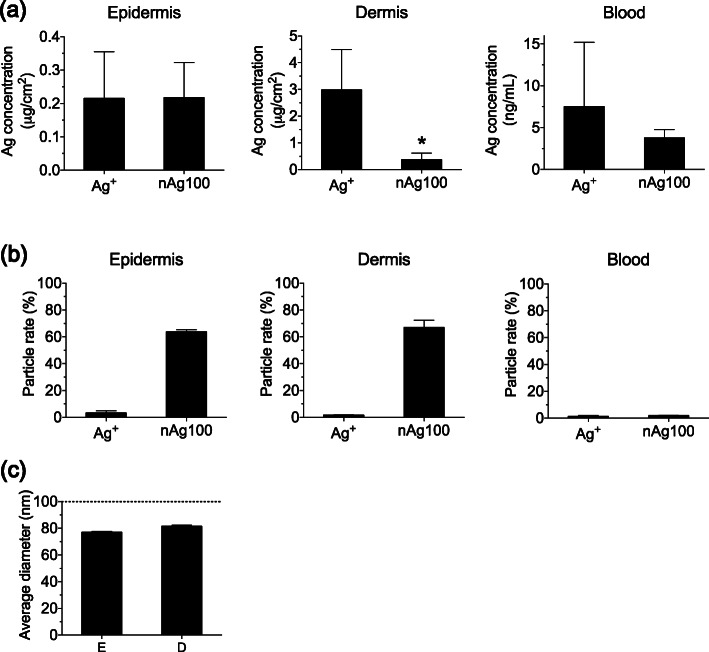
Semi-quantitative physical property analysis of nAg100 and Ag^+^ applied to mouse skin in vivo. Firstly, mouse skin was exposed to nAg100 and Ag^+^ (20 μg Ag/cm^2^). Five days after exposure, quantification, and evaluation of physical properties of Ag in epidermis, dermis, and blood were conducted via ICP-MS and sp-ICP-MS, respectively. **a** Ag concentrations and **b** particle rates detected in the epidermis, dermis, and blood. **c** The average diameter of particles detected in the epidermis and dermis. The dashed line represents the initial particle size. All data have been expressed as mean ± S.E. (*n* = 3). *p < 0.05 (Student’s *t* test)

In order to evaluate the practical application of this approach, we analyzed the quantity and physical properties of Ag in the epidermis and dermis as well as in the peripheral blood, after mouse skin was exposed to nAg100 and Ag^+^ in vivo. Five days after exposure, we separated the skin into the epidermis and dermis under optimized conditions for microwave irradiation and solubilized these using NaOH, as described in the previous section. ICP-MS analysis indicated that Ag was present in all tissues (such as the epidermis, dermis, and the blood) of all groups. In the dermis and the blood, Ag in the Ag^+^-exposed group tended to be increased, compared to that in the nAg100-exposed group (Fig. 4a). The data suggested that although both the ion and particle forms were percutaneously absorbed and distributed as previously reported [8, 9], it was easier for the ionic forms to infiltrate into deep tissue layers than it was for the particle forms.

Next, we evaluated the ratio of nAg and Ag^+^ in each sample. In the Ag^+^-exposed group, almost all Ag was detected in ionic form in the epidermis, the dermis, and the blood, indicating that Ag^+^ was percutaneously absorbed and distributed without a change in physical properties. Conversely, in the nAg100-exposed group, approximately 70% of the Ag in the epidermis and dermis was present in particle form, while the remaining Ag was ionized. Furthermore, Ag detected in the peripheral blood was almost totally ionized and the particle form was hardly detected (Fig. 4b). Finally, we evaluated the particle size in the epidermal and dermal layers, where mainly particle forms were detected. sp-ICP-MS analysis revealed that particle sizes detected in both layers were approximately 70–80 nm, corresponding to the rate at which nAg100 is ionized (Fig. 4c). These data suggest that when the skin is exposed to nAg100, it could be absorbed and distributed while being ionized.

As previously reported, the surface ligands of nanoparticles affected their absorption into the skin [25, 26]. For example, gold nanoparticles modified with an amino group were absorbed into the mouse and human skin to a greater extent than gold nanoparticles modified with carboxyl group were in ex vivo experiments [25]. Moreover, molecular dynamics analysis indicated that the skin-penetrating ability decreased from neutral hydrophobic to cationic to anionic gold nanoparticles in that order [26]. In this respect, it was inferred in this study that nAg100 was less likely to penetrate the SC in the epidermis, the initial skin barrier, because nAg100 was modified with citrate and negatively charged. Therefore, the reason for the particles being observed in the dermis and the blood could be that the particles penetrated through the pores as well as through the epidermis.

It has also been reported that the penetrability of gold nanoparticles was improved by modification with cell-penetrating peptides, such as Tat and R7 [25]. Therefore, in the future, similar modifications can be considered for nAgs in order to deliver them deeper into the skin. Furthermore, it may be necessary to reduce the size of the nAgs, because the effect of the surface modification is greater with a larger specific surface area.

## Conclusions

In this study, we developed a pretreatment approach to semi-quantitatively determine the physical properties of nAg100 in each skin layer with sp-ICP-MS. Using this approach, we showed that exposure of skin to nAg100 could result in nAg100 being ionized, absorbed, and distributed into deeper layers. Therefore, in order to understand biological responses or toxicity associated with exposure of skin to nAg100, it may be necessary to consider not only the distribution of nAg100, and its particle size, but also that of Ag^+^ from nAg100, which melts into skin tissues. Therefore, this approach shows promise as a fundamental technique that may be used for risk analysis.

## Data Availability

Data sharing is not applicable to this article as no datasets were generated or analyzed during the current study.

## Abbreviations

ENPs: Engineered nanoparticles
nAg: Silver nanoparticle
Ag^+^: Silver ions
sp-ICP-MS: Single particle inductively coupled plasma mass spectrometry
SC: Stratum corneum
nAg100: Silver nanoparticle with diameter of 100 nm
AgNO_3_: Silver nitrate
NaOH: Sodium hydroxide
HNO_3_: Nitric acid
PBS: Phosphate-buffered saline
ICP-MS: Inductively coupled plasma mass spectrometry
EDJ: Epidermal-dermal junction
